# Transcriptomics and interactomics during the primary infection of an SfNPV baculovirus on *Spodoptera frugiperda* larvae

**DOI:** 10.3389/fcimb.2023.1291433

**Published:** 2023-11-21

**Authors:** Jonatan Carmen Rangel-Núñez, Jorge E. Ibarra, Ma. Cristina Del Rincón-Castro

**Affiliations:** ^1^ Posgrado en Biociencias, Departamento de Alimentos, División Ciencias de la Vida, Universidad de Guanajuato, Irapuato, Mexico; ^2^ Departamento de Biotecnología y Bioquímica, Centro de Investigación y de Estudios Avanzados del IPN (CINVESTAV) Unidad Irapuato, Irapuato, Mexico

**Keywords:** *Spodoptera frugiperda*, transcriptomic, interactomic, baculoviruses, primary infection

## Abstract

The fall armyworm (FAW), *Spodoptera frugiperda*, has been the most devastating pest of corn as well as of other crops in America, and more recently in Africa and Asia. The development of resistance to chemical insecticides led the search for environmentally friendly biological alternatives such as baculoviruses. This study focuses on the primary infection of the baculovirus SfNPV-Ar in the FAW’s midgut epithelium, by analyzing the differential expression of transcripts in excised midguts at 6, 12, and 24 h post-infection (hpi), and predicted their interactions. Interaction of viral factors with the infected midgut tissue could alters various cellular processes, such as the apoptotic system due to the up-regulation observed of FABP at 6 hpi and of HSP90 at 24 hpi, along with the down-regulated PRX at 6 hpi and FABP transcripts between 12 and 24 hpi. Changes in transcript regulation could affect the cellular architecture of infected cells due to up-regulation of ARP 2/3 at 6 and 12 hpi, followed by down-regulation at 24 hpi. In relation to protein folding proteins, HSP90 was up-regulated at 24 hpi and PDI was down-regulated between 6 and 12 hpi. With respect to metabolism and cellular transport, AcilBP and ATPS0 were up regulated at 6 hpi and 12 hpi, respectively. In reference to transcription and translation up-regulation of RPL11 at 6 hpi and of FPN32 and RPL19 at 24 hpi was detected, as well as the down-regulation of RPL19 at 6 hpi, of PDI and RPL7 at 12 hpi, and of FABP at 24 hpi. In conclusion, gene regulation induced by viral infection could be related to the cytoskeleton and cellular metabolism as well as to oxidative stress, apoptosis, protein folding, translation, and ribosomal structure. The results presented in this work are an approach to understanding how the virus takes control of the general metabolism of the insect host during the primary infection period.

## 1 Introduction

Globally, corn production is threatened by a yield loss of up to 40% caused by the fall armyworm (FAW), *Spodoptera frugiperda* J. E. Smith (Chimweta et al., 2020). This pest is a serious problem in America (Murúa et al., 2015) and its devastating effect is expanding to Africa (Goergen et al., 2016), along with reports of its presence in Asia (Sun et al., 2021) and Oceania (Liu et al., 2020). The control of *S. frugiperda* is mainly based on the use of chemical insecticides and *Bacillus thuringiensis* toxins; unfortunately, development of resistance to these alternatives is frequent (Horikoshi et al., 2016).

Among the biological control alternatives against *S. frugiperda*, baculoviruses, either nuclear polyhedron viruses (NPVs) or granuloviruses (GVs) have a great potential because the possible development of resistance towards these agents is much lower than the other alternatives. Additionally, baculoviruses are insect-specific viruses and completely safe to humans and other mammals. Baculoviruses are double-stranded DNA viruses, with genomes ranging from 80 to 180 kb, and classified into four different genera: *Alphabaculovirus*, *Betabaculovirus*, *Deltabaculovirus*, and *Gammabaculovirus* (Jehle et al., 2006). Different strains of baculoviruses isolated in the American continent, the native land of FAW, have been reported with the potential to control it. These isolates show significant differences in their mean lethal concentration (LC_50_s), indicating unique molecular features for each strain, which impact on their virulence, and this may be related to the fact that baculoviruses constantly evolve and modulate their interaction with their host during replication and spread, encoding multifunctional genes that interact with the host gene expression, modifying cellular processes to improve their replication and spread (Escribano et al., 1999; Barreto et al., 2005; Vásquez et al., 2006; Yasem de Romero et al., 2009; Rangel-Núñez et al., 2014).

Various quantitative or semi-quantitative analyses based on the detection of differentially expressed genes have been used to characterize baculoviruses and their interaction with the host. It has been reported that viral infection can affect the regulation of host gene expression in the primary tissue (midgut epithelium) and in the rest of susceptible tissues in insects, such as hemocytes, epidermis, tracheal matrix, fatty tissue (Bao et al., 2009; Bao et al., 2010; Jakubowska et al., 2010; Liu et al., 2010; Donly et al., 2014; Donly et al., 2016; Wang et al., 2016; Yu et al., 2016; Xing et al., 2017). Among the cellular effects observed in insects during baculovirus infection, a modulation of gene expression systems has been found as the viral infection progresses, with the activation and deactivation of defense systems, such as the production of reactive oxygen species (ROS) (Li et al., 2012), up-regulation of chaperones, and activation of the unfolded protein response (UPR) system (Koczka et al., 2018), as well as modulation of apoptotic systems driven by caspases, caused by the expression of viral anti-apoptotic proteins (Schultz and Friesen, 2009; Nagamine, 2022).

Furthermore, changes in insect cell architecture have been observed, induced by baculovirus infection, with modifications in the expression of cytoskeleton-related genes (Li et al., 2022). Also, transport of intracellular lipids in insects is modulated by baculovirus infection (Nagamine et al., 2019), which affects their cell cycle, causing its arrest due to the expression of viral genes (Xiao et al., 2021). Previously we have shown high virulence of the SfNPV-Ar strain in *S. frugiperda*, as compared with other strains, namely SfNPV-Fx and SfNPV-Ho (Rangel-Núñez et al., 2014), which is why we have used SfNPV-Ar to provide evidence for host differential gene expression using a transcriptomic approach including some interactomics for those differentially expressed genes.

## 2 Materials and methods

### 2.1 Rearing of *S. frugiperda* and infection with SfNPV-Ar

The FAW colony was established in the Food and Plant Biotechnology Laboratory at the University of Guanajuato, Mexico, from field-collected specimens. The rearing procedure (Zanella-Saenz et al., 2022) was carried out as follows. Larvae were maintained in a meridic diet containing 100 mL distilled water, 12.5 g bacteriological agar, 120 g corn flour, 50 g yeast, 5 g wheat germ, 25 g ground corn spike, 2.5 g sorbic acid, 5 g ascorbic acid, 3.125 g methylparaben, 8.75 g Wesson’s salt mixture, 62.5 g soybean, 3.125 mL formaldehyde 37%, 0.75 mg streptomycin, and18.75 g Vanderzant vitamin mixture. They were maintained under insectarium conditions (60 ± 10% relative humidity, 26 ± 2°C and 16:8 h light: dark photoperiod) in an environmental chamber.

The origin, characterization, and virulence of SfNPV-Ar was previously reported by Rangel-Núñez et al. (2014), as mentioned above. To establish the optimum OB concentration able to discriminate the different infections stages in FAW larvae, different concentrations ranging from 5x10^5^ to 80x10^5^ OB/larva, in a 10% sucrose solution and 0.1% vegetable dye (W/V), were used to infect 40 3^rd^ instar FAW starved larvae with 2 µl of each suspension, as reported earlier (Hughes and Wood, 1981). The lowest tested dose showing 100% mortality was chosen as it showed all the symptoms of a disease caused by baculovirus, including the death of the insects and the extraction of the highest amount of RNA from host cells exposed to virions interfering in cell gene regulation during the primary infection.

### 2.2 Removal of FAW midguts

**Figure 1:**
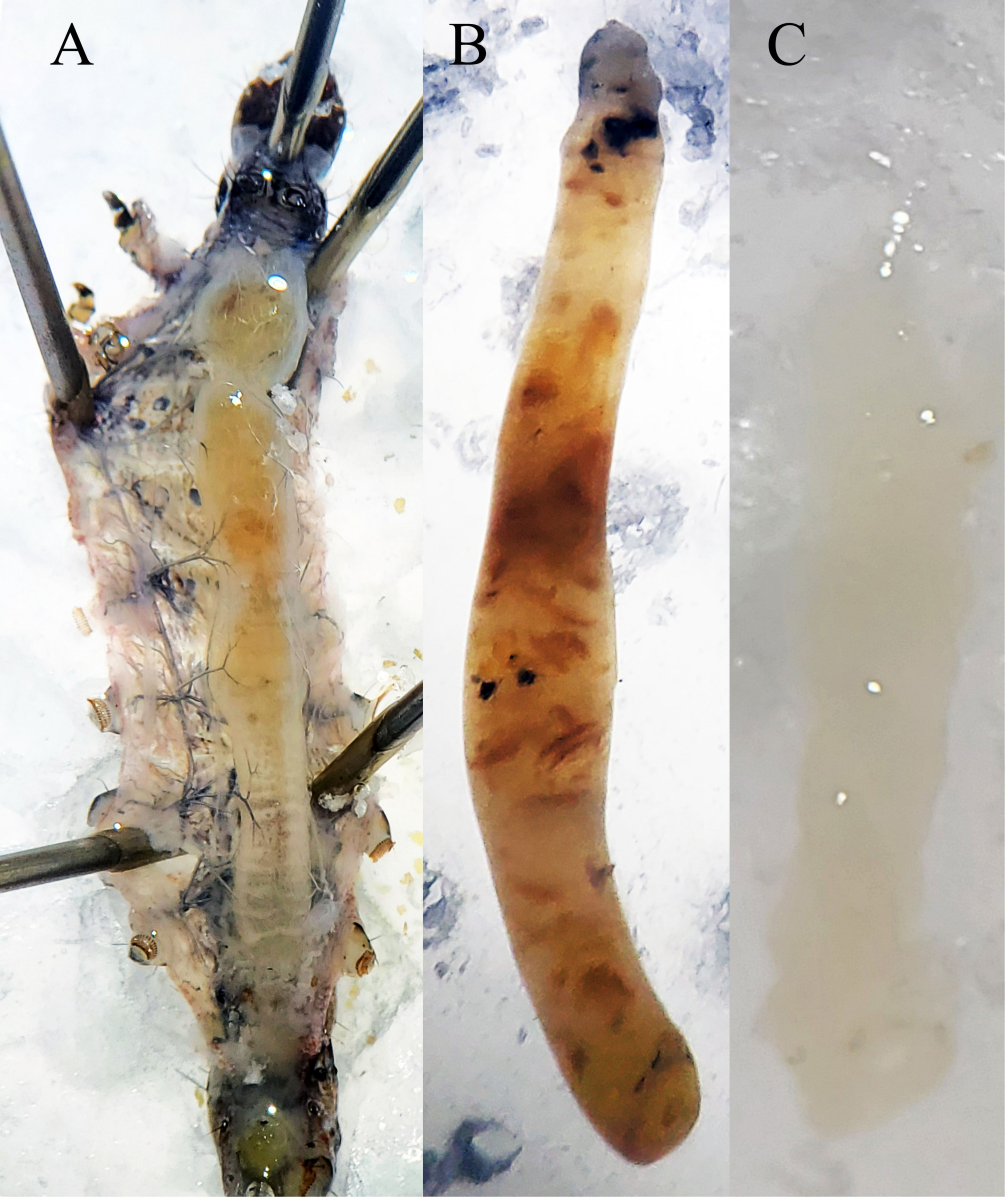
Removal of the midgut from a FAW 3^rd^ instar larva. **(A)** Initial dissection of the larva by exposing the midgut through a longitudinal ventral cut; **(B)** Midgut food content surrounded by the peritrophic matrix, extracted from the excised midgut, previously dislodged from peripheral tissues and tracheae; **(C)** Emptied midgut ready to be processed.

Midguts were removed from infected larvae at 6, 12, and 24 hours postinfection (hpi) and an equivalent number of untreated insects were used as control for each period. For each post-infection period, 50 3^rd^ instar larvae were infected at a concentration of 60x10^5^ OB/larva, as described above. Therefore, 300 infected midguts were removed by microdissection under a stereomicroscope (Carl Zeiss Stemi DV4) (**Figure 1**), following the technique described in Shrestha et al. (2019). Briefly, larvae were immobilized in a dissection tray filled with saline by pinning the head and the posterior end. Then, a ventral longitudinal section was made with microdissection scissors, exposing the whole midgut, and dislodging the gut from peripheral tissues, including tracheae and tracheoles. Midguts were excised from the body with the microdissection scissors and the food content was removed by carefully pulling the peritrophic matrix out of the midgut (**Figure 1**). Emptied midguts (midgut epithelium) were immersed in RNAlater solution (Invitrogen) and stored at 4°C.

### 2.3 RNA extraction

For total RNA extraction, 50 midguts from either infected or control larvae were used for each mentioned post-infection period. They were centrifuged at 2,000 rpm for 30 min at 4°C, the supernatant was discarded, and the RNA was extracted from midguts using TRIzol™ Reagent (Invitrogen) following the supplier’s recommendations. The RNA was stored in RNase-free water at -20°C and its quality was determined according to Wieczorek et al. (2012).

### 2.4 cDNA synthesis

For cDNA synthesis, 1 µg of the RNA extracted from midguts of infected and control larvae was used. Each sample was treated with 1 U of DNase I (Invitrogen) for 10 min at room temperature, and the reaction was inactivated with 5 µL of 25 mM EDTA at 65°C for 20 minutes. The first chain (ss cDNA) was synthesized for each condition with 1 µL of CDS oligonucleotides (dT) 10 mM and 1 µL of Smart IIA 10 mM (**Table 1**). Reactions were incubated for 1 min at 70°C, 1 min at 68°C, and 1 min at 65°C. Samples were mixed with 6 µL of the specific enzyme buffer Super Script RT III 5X (Invitrogen), 1 µL of DTT (0.1 mM), 1 µL of dNTP’s (10 mM), 400 units of Super Script RT III (Invitrogen), to a volume of 30 µL with sterile distilled water (SDW). Reactions were incubated at 42°C for 40 minutes and then inactivated at 70°C for 20 minutes. For the double-stranded cDNA (ds cDNA) for each condition, 1 µL of the newly synthesized ss cDNA, 10 µL of Super Fi II Solution (5x) (Invitrogen), 1 µL of PCR IIA oligonucleotide (10 mM) (**Table 1**), 1 µL of dNTPs (10 mM), and 1 unit of Platinum Super Fi II DNA polymerase (Invitrogen) were used. Final volume was adjusted to 50 µL with SDW and PCR was performed under the following conditions: 94°C for 5 minutes, 25 cycles of 94°C for 30 sec, 65°C for 45 sec, 68°C for six min, and a final cycle of 72°C for 10 min.

**Table 1:** Primers used for the construction of the substractive library (V: A, C or G; X: undisclosed base).

Primer	Sequence
CDS (dT)	5’–AAGCAGTGGTATCAACGCAGAGTACTN(30)V–3’
Smart IIA	5’–AAGCAGTGGTATCAACGCAGAGTACXXXXX–3’
PCR IIA	5’– AAGCAGTGGTATCAACGCAGAGTAC–3’
Adapter 1	5’-CTAATACGACTCACTATAGGGCTCGAGCGGCCGCCCGGG CAGGTGGCCCGTCCA-3’
Adapter 2	5’-CTAATACGACTCACTATAGGGCAGCGTGGTCGCGGCC GAGGTGCCGGCTCCA-3´
PCR primer I	5´- CTAATACGACTCACTATAGGGC-3´
Nested PCR primer I	5´-TCGAGCGGCCGCCCGGGCAGGT-3´
Nested PCR primer 2	5´-AGCGTGGTCGCGGCCGAGGT-3´

### 2.5 Hybridization of cDNA library under subtractive suppression conditions

For the construction of the cDNA library under subtractive suppression conditions (SSC), the protocol outlined by Diatchenko et al., 1996 was followed with some modifications. Firstly, 100 µg ds cDNA synthesized from the total RNA extracted from infected and control FAW larvae were digested with 1 U RsaI (Invitrogen), 2 µL 10X Anza Buffer (Invitrogen), and an adjusted to final volume of 20 µL with SDW at 37°C for 3 hours. The reaction was purified with the PureLink™ PCR Purification Kit (Invitrogen). Subsequently, Adapters 1 and 2 (**Table 1**) were ligated by mixing 100 ng of digested ds cDNA (“tester”), five units of T4 DNA ligase (Invitrogen), 3 µL 10X buffer, 1 µL of Adapter 1 (10 mM), to a final volume of 30 µL with SDW at 16°C for 16 hours. The enzyme was inactivated with 1 µL EDTA 200 mM pH 8.6 and incubated at 70°C for 5 minutes. The same protocol was followed for adapter 2.

Then, the first hybridization was carried out. For genes up-regulated at the three tested post-infection periods, digested ds cDNA from the uninfected tissue (“driver”) was mixed with the “tester” containing Adapter 1 at a 30:1 ratio, along with a volume of the Hybridization Buffer (HB) (50 mM HEPES pH 8.3, 0.5 M NaCl, 0.02 mM EDTA pH 8.0, 10% PEG 800). The same was repeated for the “tester” containing Adapter 2, separately. For genes down-regulated by the infection, the same procedure was followed, but the ds cDNA from the uninfected tissue with adapter 1 and 2 was used as the “tester”, and the digested ds cDNA from the infected tissue was used as the “driver”. The above samples were incubated at 98°C for 90 seconds, followed by 68°C for 10 hours. After this period, the 2^nd^ hybridization was performed, that is, the hybridizations with adapters 1 and 2 were mixed in the same tube at 68°C, and 150 ng of the corresponding “driver” was added, previously denatured at 98°C for 2 minutes, along with a volume of HB. The reaction was kept at 68°C for another 12 hours. Afterwards, 200 µL dilution buffer (20 mM HEPES pH 8.3, 50 mM NaCl, 0.2 mM EDTA) was added. The mixture was heated to 72°C for 7 minutes and stored at -20°C.

Following the hybridizations, two PCRs were performed. First, a reaction mixture was prepared with 1X Super Fi II Solution (5X) (Invitrogen), 1 µL dNTPs (10 mM), 1 unit of Platinum Super Fi II DNA polymerase (Invitrogen), 1 µL PCR primer I (**Table 1**) (10 mM), 1 µL of the second hybridization, and the volume was completed to 50 µL with SDW. The PCR conditions were 72°C for 5 minutes, 94°C for 1 minute, 35 cycles of 94°C for 25 seconds, 56°C for 30 seconds, 68°C for two minutes, and one cycle of 72°C for seven minutes. This PCR was diluted 1:30 with the dilution buffer and stored at -20°C. For the second PCR, a reaction mixture was prepared with 1X Super Fi II Solution (5X) (Invitrogen), 1 µL dNTPs (10 mM), 1 unit of Platinum Super Fi II DNA polymerase (Invitrogen), 1 µL Nested PCR primer 1 (10 mM) (**Table 1**), 1 µL Nested PCR primer 2 (10 mM) (**Table 1**), 1 µL of the previous PCR dilution, and SDW to 50 µl. PCR was carried out under the following conditions: 94°C for 1 minute, 25 cycles of 94°C for 30 seconds, 58°C for 45 seconds, 68°C for two minutes, and one cycle of 72°C for 7 minutes.

Amplicons from the second PCR were ligated into the PCR4 TOPO TA Cloning Kit vector (Invitrogen), following the manufacturer’s recommendations. Constructs were used to transform *E. coli* Top10 cells by thermal shock as follows: 1 µL of the previous ligation product was added to competent cells, which were incubated on ice for 30 minutes followed by a thermal shock at 42°C for 1 minute. 1 mL of LB broth base (Invitrogen) was added and incubated at 37°C and 100 rpm for an hour. Colonies grew up in LB agar complemented with ampicillin (100 ng/mL) at 37°C overnight. Subsequently, individual colonies were selected and inoculated in 3 ml of LB broth with ampicillin (100 ng/ml) at 37°C and 150 rpm overnight. Afterwards, plasmid DNA was extracted by alkaline lysis as reported by Kotchoni et al. (2003) and sequenced (MACROGEN, Inc. South Korea), using the Sanger technique. Sequences were assembled with the SeqMan II software (DNASTAR). Identification, annotation, and function were carried out using the BLASTn platforms under the following parameters: Standard databases, Nucleotide collection (nr/nt) optimization: Highly similar sequences MegaBlast, Max target sequences: 100, Expected threshold: 0.05, word size: 28, Max matches in a query range: 0, Match/mismatch: 1-2, filter: Low complexity regions, Mask: Mask for lookup table only; the Kyoto Encyclopedia of Genes and Genomes (KEGG), and protein-protein interactions were estimated by the STRING database system v. 11.5 (https://string-db.org). Subsequently, specific oligonucleotides were designed to obtain the ORFs from the ds cDNA from the different conditions and register in the Genetic Database (GenBank) of the NHI (National Institute of Medicine, USA).

The procedure to detect any change on the expression of host genes during the primary infection is described as follows. To determine if a transcript was up- or down-regulated after the primary infection, Southern blot analysis was used with the DIG DNA Labeling and Detection kit (Roche), following the manufacturer recommendations. Therefore, 25 µg plasmidic DNA from each of the subtractive libraries’ fragments were fixed in duplicate on nitrocellulose membranes. Each membrane was hybridized with digoxigenin deoxyuridine triphosphate (dUTP) labeled cDNA obtained from the three mentioned periods, from infected and uninfected midgut samples. Higher hybridization with cDNA from uninfected midguts indicated down-regulated transcripts, while higher hybridization with cDNA from infected midguts indicated up-regulated transcripts.

### 2.6 Protein interaction analysis

To predict biological functions, location, and interactions of differentially expressed genes, the open reading frame of the identified genes was first identified and translated into their amino acid sequence using the Unipro UGEN program. This sequence was analyzed using the STRING database system (https://string-db.org/) version 11.5. Protein interactions were classified into clusters according to their function, providing a prediction of the effect on down-regulated or up-regulated transcripts from the infection at different post-infection times.

## 3 Results

### 3.1 Infection of FAW larvae

Six doses of SfNPV-Ar (5x10^5^, 10x10^5^, 20x10^5^, 40x10^5^, 60x10^5^, and 80x10^5^ OBs/larva) (**Table 2**) were tested to establish the working concentration to analyze genes regulated during the primary infection process. Based on the infection showing high mortality rate, 60x10^5^ OBs/larva was selected as working concentration, to obtain the highest amount of host cell RNA, based on the infection progression in larvae treated with this dose. Emptied midguts were successfully obtained and processed. No morphological differences were observed between infected and control larvae, since no OBs formed at this stage i.e., 6, 12 & 24 hrs after primary SfNPV-Ar infection (Blissard and Rohrmann, 1990) (**Figure 1**).

**Table 2:** Preliminary test to select the working dose to optimize the extraction of mRNA.

Dose (OB/larva)	Total tested larvae	Mortality (%) 7 dpi
0	40	0 ^a^
5x10^5^	40	10 ^d^
10x10^5^	40	20 ^d^
20x10^5^	40	65 ^c^
40x10^5^	40	75 ^c^
60x10^5^	40	100 ^b^
80x10^5^	40	100 ^b^

Different numbers at mortality levels indicate statistical difference (P<0.05).

### 3.2 RNA extraction

**Figure 2:**
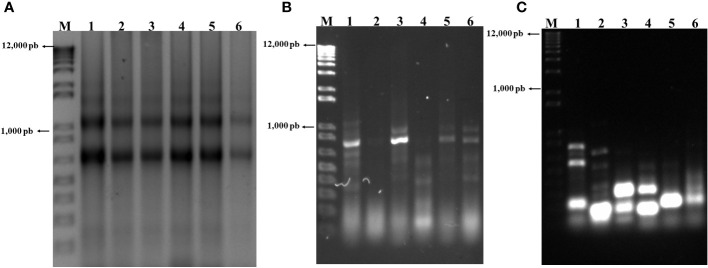
**(A)** RNA extracted from midguts of a set of uniform, selected group of FAW larvae. **(A)** Lanes 1, 3, and 5, RNA from control larvae 6, 12, and 24 h post selection (hps), respectively; lanes 2, 4, and 24, RNA from infected larvae 6, 12, and 24 hpi. **(B)** Lanes 1, 3, and 5, ds cDNA from control larvae at 6, 12, and 24 hps; lanes 2, 4, and 6, ds cDNA at 6, 12, and 24 hpi. **(C)** Lanes 1, 3, and 5, transcripts down-regulated by infection at 6, 12, and 24 hpi, respectively; lanes 2, 4, and 6, transcripts up-regulated by infection at the same periods.

RNA was extracted from infected and uninfected midguts from a uniform set of larvae and visualized on 1.5% agarose gels (**Figure 2A**). RNA quality was determined based on the criteria described by Wieczorek et al. (2012), revealing a uniform smear across all lanes in **Figure 2A**, with the presence of 18S and 28S ribosomal RNAs at 750 and 1200 bp, respectively. Absorbance at 260 nm was recorded below 25 for all samples, showing RNA stability. The 260/280 nm ratio yielded values between 1.7-2.0 under all conditions.

### 3.3 cDNA synthesis

Using the RNA from the previous step, ds cDNA was synthesized and visualized as shown in **Figure 2B**. From this ds cDNA, all the transcripts up- and down-regulated by the infection at 6, 12, and 24 hpi were obtained (**Figure 2C**, **Table 3**). Differences in banding patterns between infected and control larvae were observed, which may be influenced by the viral infection and its effects on gene expression in the infected tissue at various times (**Figures 2B, C**). The fragments obtained from the reaction, both down- and up-regulated by the infection at different infection periods (**Figure 2C**), were ligated into the pCR4-TOPO TA vector and amplified in *E. coli* TOP 10 cells.

**Table 3:** Southern blot analysis of the differential genes obtained at 6, 12 and 24 hpi, by the infection of SfNPV baculovirus on *S. frugiperda* larvae.

Hours post-infection	Affected system	Transcript (Uniprot code)	Regulation	Immunodetection
**6 hpi**	Apoptosis	Peroxiredoxin (Jafrac1)	D-R	6 h control 6 hpi 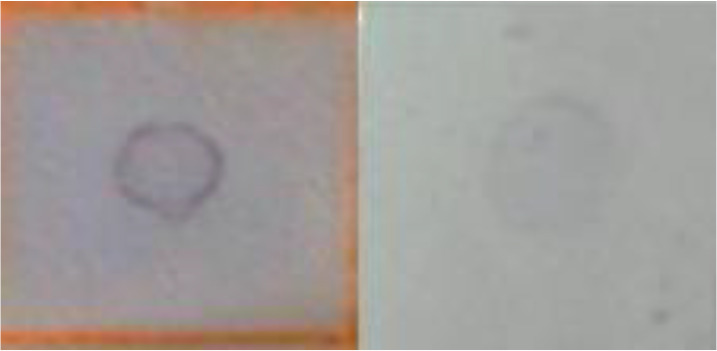
		Fatty acid binding protein (LOC732863)	U-R	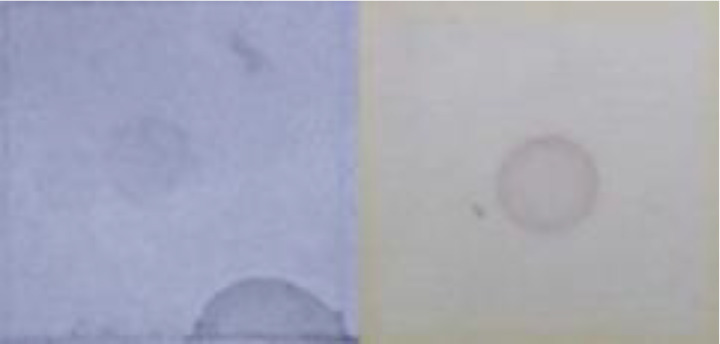
	Cytoskeleton	Actin related protein 2/3 complex subunit 2 (Arpc2)	U-R	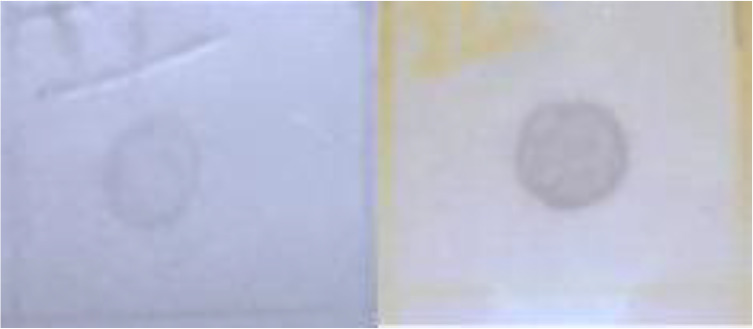
	Protein folding	Muscle-specific protein 20 (MEP20)	D-R	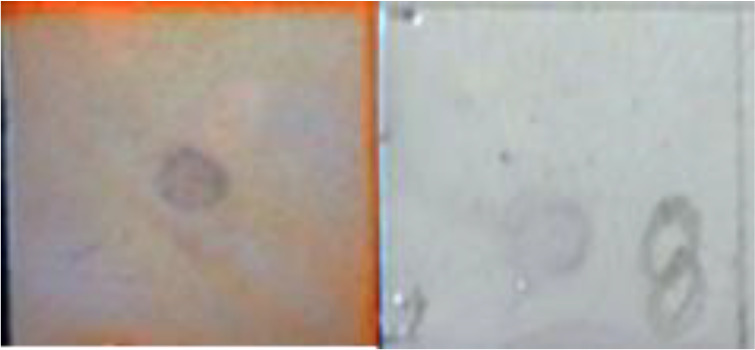
		Protein disulfide isomerase (A0A2A4JF99)	D-R	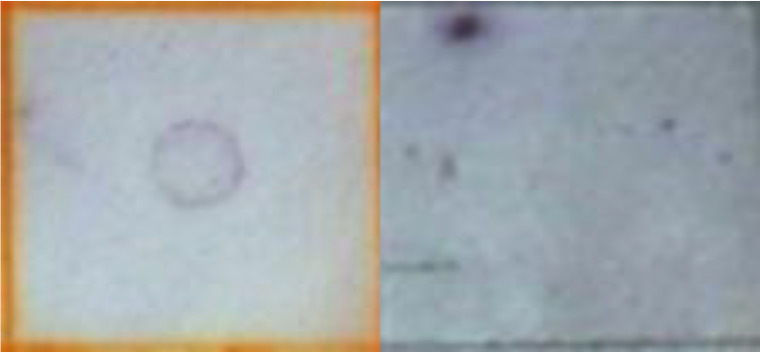
	Lipid metabolism and transport	AcylCoA binding protein (ACBP-like)	U-R	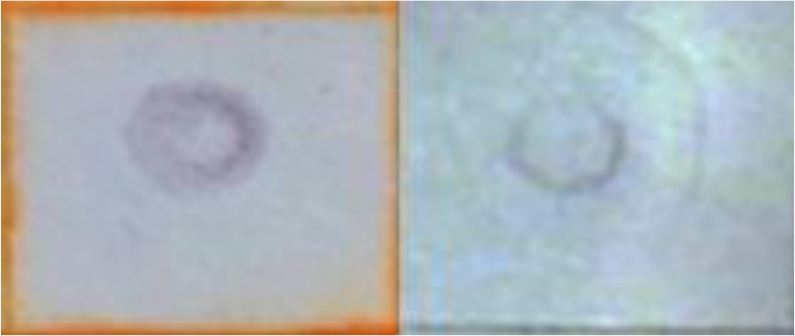
	Transcription and translation	60S ribosomal protein L19 (A0A2A4IZU1)	D-R	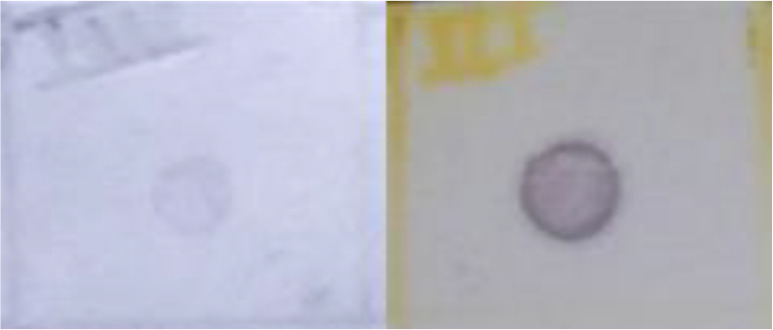
		Ribosomal protein L11 (RpL11)	U-R	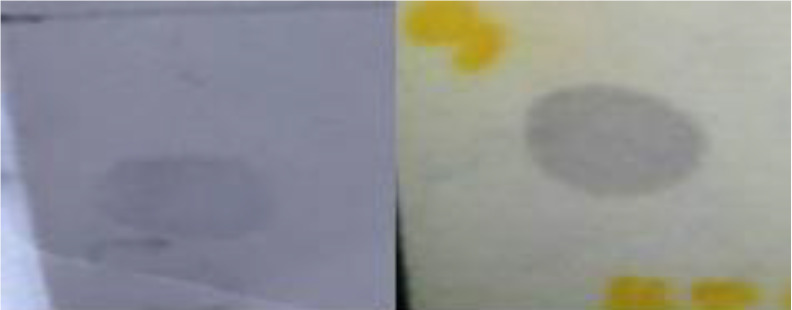
**12 hpi**	Apoptosis	Fatty acid binding protein (LOC732863)	D-R	12 h control 12 hpi 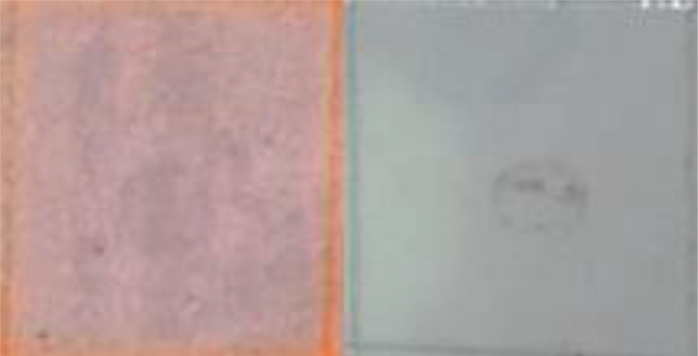
	Cytoskeleton	Actin related protein 2/3 complex subunit 2 (Arpc2)	U-R	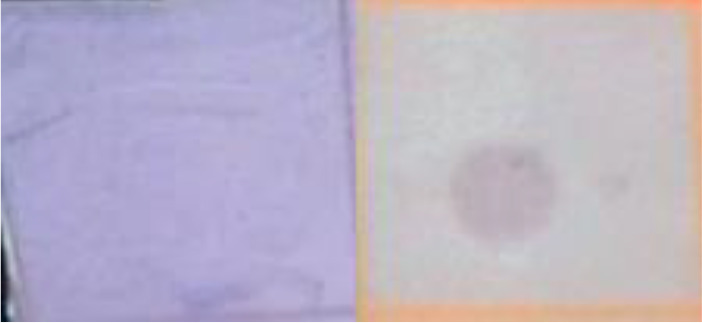
		MG17 protein (MG17)	D-R	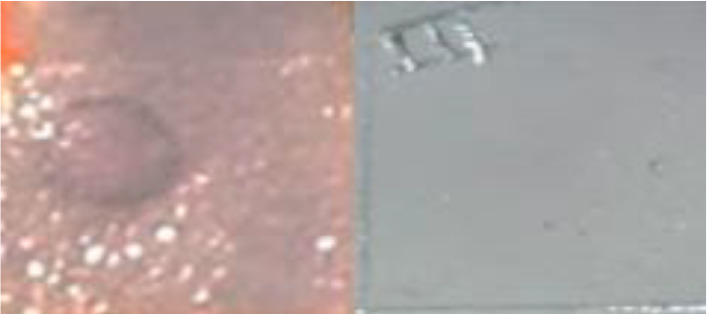
	Protein folding	Protein disulfide isomerase (A0A2A4JF99)	D-R	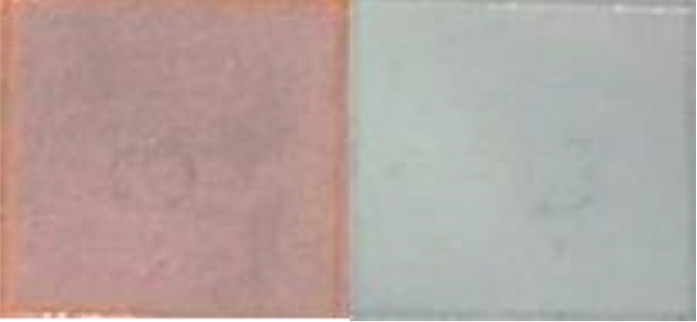
	Lipid metabolism and transport	ATP synthase subunit O mitochondrial (A0A2A4J874)	U-R	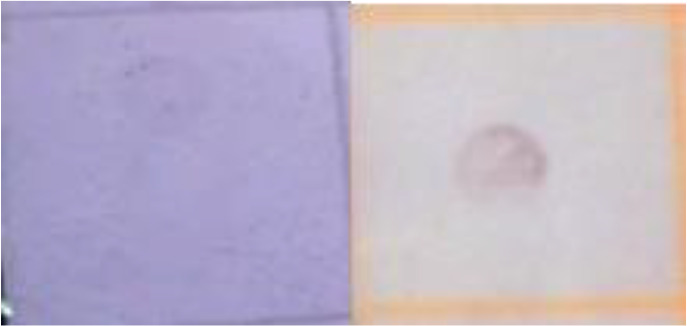
	Transcription and translation	Ribosomal protein L7 (RpL7)	D-R	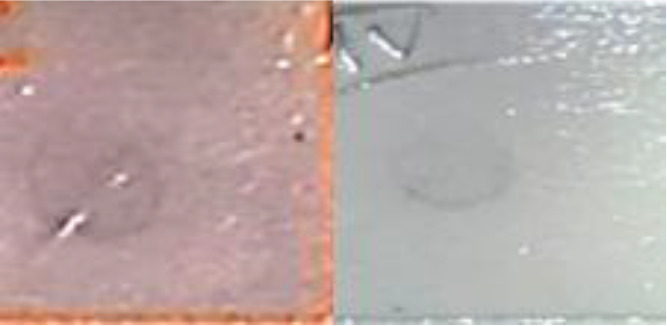
**24 hpi**	Apoptosis	Fatty acid binding protein (LOC732863)	D-R	24 h control 24 hpi 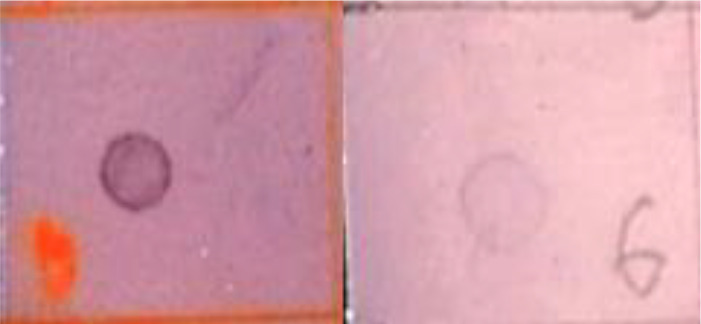
	Cytoskeleton	Actin related protein 2/3 complex subunit 2 (Arpc2)	D-R	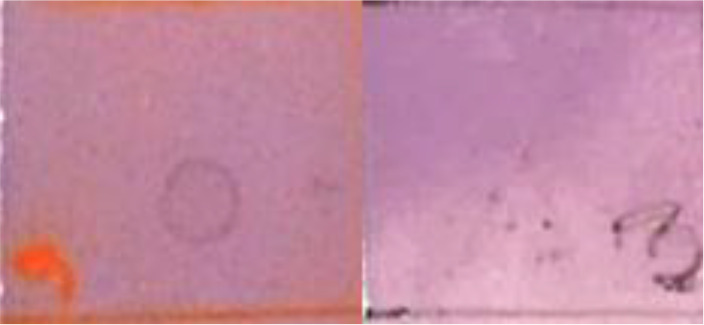
		Acidic leucine rich nuclear phosphoprotein 32 (Anp32a)	U-R	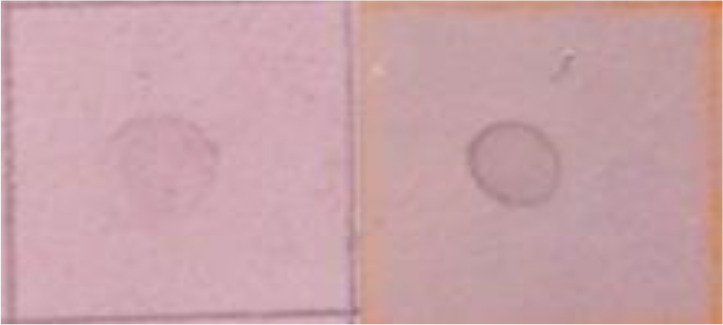
	Protein folding	HSP90 (Hsp90)	U-R	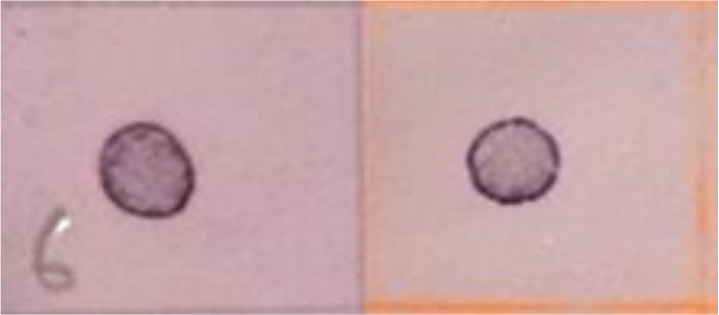
	Transcription and translation	Ribosomal protein L7 (RpL7)	D-R	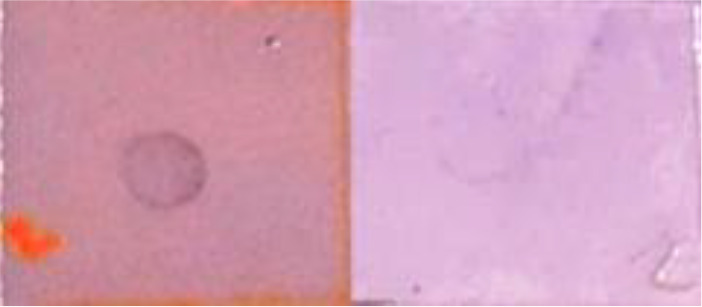
		60S ribosomal protein L19 (A0A2A4IZU1)	U-R	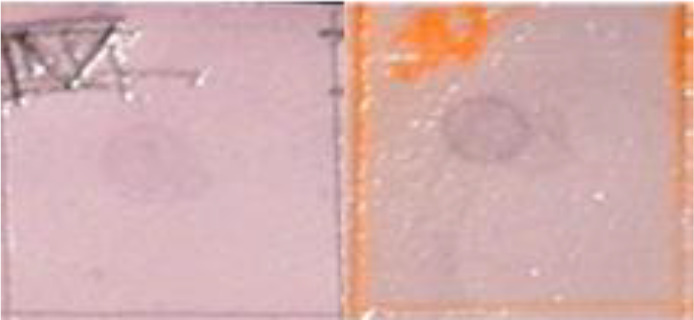

U-R, Up-regulated; D-R, Down-regulated; hpi, hours post infection.

### 3.4 Hybridization of cDNA library under subtractive suppression conditions

**Figure 3:**
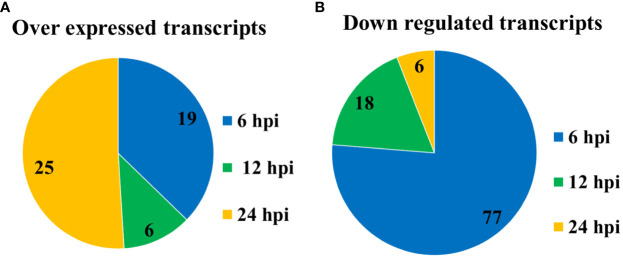
Number of transcripts regulated by the primary infection of the SfNPV-Ar baculovirus. **(A)** Down-regulated; **(B)** up-regulated.

Plasmid DNA from selected colonies obtained from the previous step were subjected to Southern Dot Blot analysis (**Table 3**). As a result, 151 clones were obtained which showed differential transcript fragments belonging to 14 different genes. From these, 101 clones were identified as down-regulated by the infection, and 50 clones were identified as up-regulated during the whole primary infection period. At 6 hpi, the largest number of down-regulated transcripts (77) was observed (**Figure 3A**), while 19 were up-regulated (**Figure 3B**). At 12 hpi, a reduced number of down-regulated transcripts (18) was detected (**Figure 3A**), with only 6 up-regulated transcripts affected by the infection (**Figure 3B**). Finally, at 24 hpi, only 6 transcripts were down-regulated (**Figure 3A**), but a significant number of up-regulated transcripts (25) were detected at this last post-infection period (**Figure 3B**).

**Figure 4:**
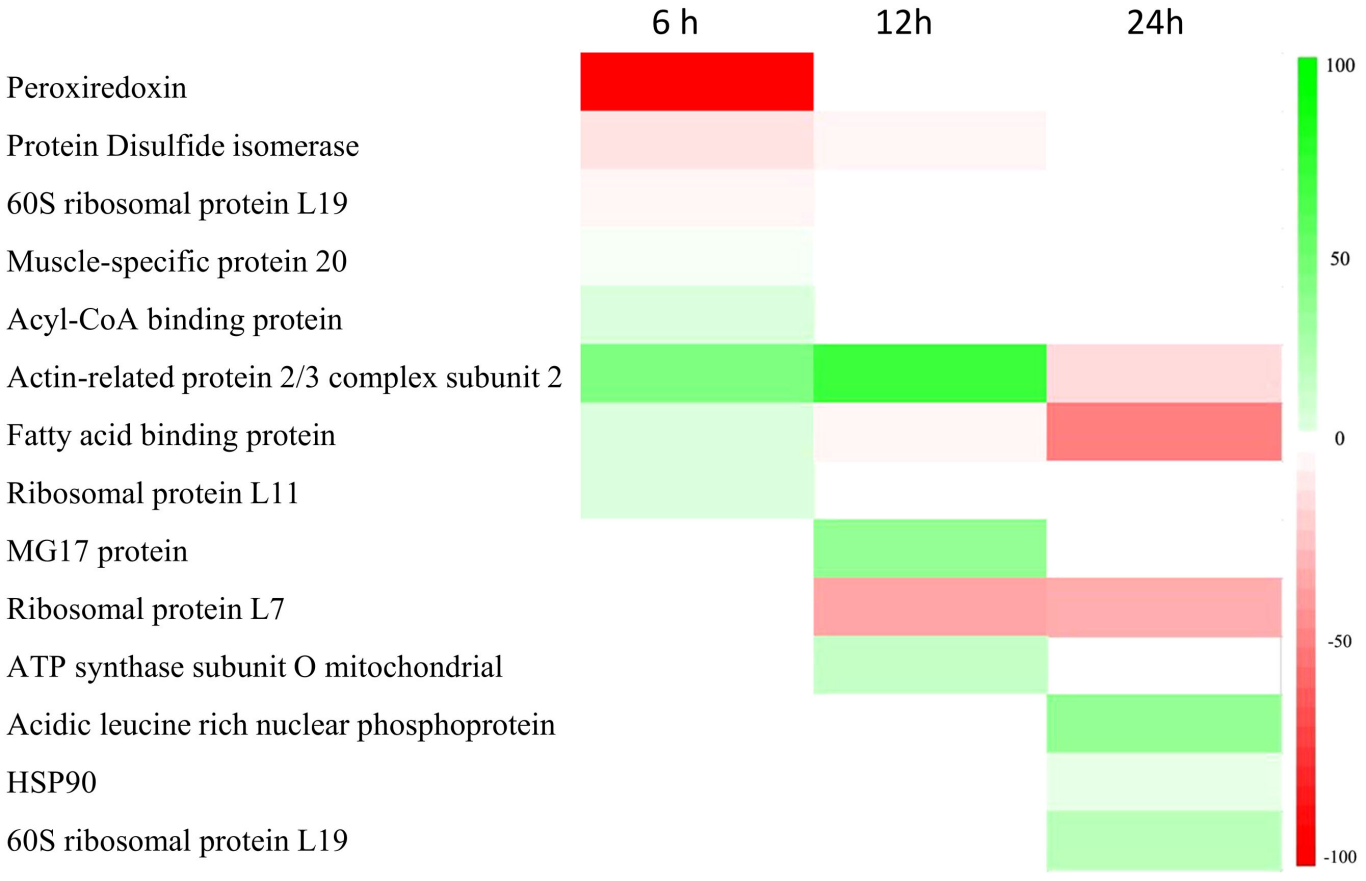
Heatmap with the 14 genes and the frequency (percentage) they are down- or up-regulated at a given infection period.

Transcripts were identified through Southern blot analysis (**Table 3**), sequencing and annotation of the possible proteins they encode (**Table 4**). At 6 hpi, transcripts for proteins such as peroxiredoxin (PRX) (71.3% of the total identified transcripts), disulfide isomerase (PDI) (4.3%), 60S ribosomal protein L19 (RPL19) (7.1%), and muscle-specific protein 20 (MEP20) (7.1%) were down-regulated (**Tables 3**, **4**). For the up-regulated RNA transcripts in the insects at 6 hpi, proteins like acylCoA binding protein (AcilBP) (16.66%), actin-related protein 2/3 complex subunit 2 (ARP 2/3) (50%), fatty acid binding protein (FABP) (16.66%), and ribosomal protein L11 (RPL11) (16.66%) were identified (**Tables 3**, **4**). At 12 hpi, the down-regulated transcripts corresponded to MG17 protein (MG17) (42.8%), ribosomal protein L7 (RPL7) (35.7%), PDI (7.1%), an uncharacterized protein (UCP) (7.1%), and FABP (7.1%) (**Tables 3**, **4**). For the up-regulated transcripts at this post-infection period, proteins such as ARP 2/3 (75%) and ATP synthase subunit O mitochondrial (ATPS0) made up to 25% of the transcripts (**Tables 3**, **4**). At 24 hpi, the down-regulated transcripts were from proteins ARP 2/3 (16.6%), FABP (50%), and RPL7 (33.33%) (**Table 3**). Finally, at the same post-infection period, the up-regulated transcripts corresponded to RPL19 (28.5%), acidic leucine rich nuclear phosphoprotein 32 (FPN32) (57.1%), and heat shock protein 90 (HSP90) (14.2%) (**Tables 3**, **4**). A heatmap (**Figure 4**) shows the regulation of all 14 genes and the frequency (percentage) they are down- or up-regulated at a given infection period, as a proportion of total transcript fragments per period.

**Table 4:** Identification of differential genes during the primary infection of FAW larvae by SfNPV-Ar.

Stage	Frequency of identified fragments and corresponding accession #	Identity (%)	Stage	Frequency of identified fragments and corresponding Accession #	Identity (%)
6 hpi	Peroxiredoxin, 71.3% XM_022973939.1	98	12 hpi	Uncharacterized protein, 7.1% XM_022959916.1	94.65
	Disulfide isomerase, 14.3% XM_022974152.1	97.3		Fatty acid binding protein, 7.1% JF896323.1	86
	60S ribosomal protein L19, 7.1% XM_022965735.1	5.83		Actin-related protein 2/3 complex subunit 2, 75% XM_026890508.1	98
	Muscular protein 20, 7.1% XM_022959000.1	92.54		ATP synthase subunit O mitochondrial, 25% XM_022972498.1	91.77
	Acyl-CoA binding protein, 16.66% XM_022964505.1	91	24 hpi	Actin-related protein 2/3 complex subunit 2, 16.6% AHV90275.1	94.12
	Actin-related protein 2/3 complex subunit 2, 50% XM_026890508.1	98		Fatty acid binding protein, 50% JF896323.1	92
	Fatty acid binding protein, 16.66% JF896323.1	87.38		Ribosomal protein L7, 33.3% AY072288.1	97
	Ribosomal protein L11, 16.66% AF400182.1	100		60S ribosomal protein L19, 28.5% XM_022965735.1	96
12 hpi	MG17 protein, 42.8% JF964954.1	85.12		Acidic leucine rich nuclear phosphoprotein 32, 57.1% XM_022958786.1	93.22
	Ribosomal protein L7, 35.7% AY072288.1	98.68		HSP90, 14.2% HM046610.2	86
	Disulfide isomerase, 7.1% XM_022974152.1	95.45			

### 3.5 Identification of transcripts obtained in the SSC

Using the primers shown in **Table 5**, ORFs of the previously identified genes, derived from the SSC transcripts, were amplified. Sequences for each gene were obtained and registered in the NIH genetic database (GenBank) (**Table 5**). For PRX, a 447 bp sequence was amplified (No.OR103221); for PDI, a 648 bp sequence (No.OR103219) was amplified; for AcilBP a 246 bp sequence (No.OR103217) was obtained; for MEP20 a 345 bp sequence was amplified (No. OR103224), for ARP 2/3 a 708 bp sequence was produced (No. OR103216), for RPL11 a 549 bp sequence (No. OR103226) was obtained, for RPL7 a 645 bp sequence was identified (No. OR103225), for FABP a 312 bp sequence was obtained (No.OR103222), for UCP an 819 bp sequence was identified (No. OR103227), for ATPS0 a 210 bp sequence (No.OR103218) was determined, for MG17 a 363 bp sequence was obtained (No.OR103223), and finally, for FPN32 a 255 bp sequence was identified (No. OR103220).

**Table 5:** Primers designed to amplify ORFs of differential genes from ds cDNA.

Gene	Primers	Amplicon size (pb)	Accession number
PRX	D: 5´-ATGCCTCTCCAGCTGACC-3´ R: 5´-TTAGTTGGCGTCGATGAAGTA-3´	447	OR103221
PDI	D: 5´-ATGAGAGTTATCTTATTTACG-3´ R: 5´-TTATAACTCGTCTCTGGATGG-3´	648	OR103219
ARP 2/3	D: 5´-ATGATCTTACTCGAGATCAAT-3´ R: 5´-TCAGTCTCGCCTCACAAA-3´	708	OR103216
AcilBP	D: 5´-ATGTCTCTCGACGAGCAA-3´ R: 5´-TTATGCGTATTTAGGGGCTAG-3´	246	OR103217
FABP	D: 5´-ATGGCTTTCCTTGGTAAAACT-3´ R: 5´-TTATGCAGCTTTGTAGAATCTC-3´	312	OR103222
ATPS0	D: 5´-ATGTCGGTCATAAAGGGAAAT-3´ R: 5´-TTAAACWGCAGCACTGATGAG-3´	210	OR103218
MG17	D: 5´-ATGAAATCCTTCGTTGCCCT-3´ R: 5´-TTAGTTGAGTTCATCGGGGAG-3´	363	OR103223
MEP 20	D: 5´-ATGCCTGGACGTCCTATTT-3´ R: 5´-TCACTTGCCGAGAATGATTTT-3´	345	OR103224
FPN32	D: 5´-ATGAGCACCAATGAAAATAAC-3´ R: 5´-TTACGCATCCTCCTCCTC-3´	255	OR103220
RPL7	D: 5´-ATGGTTGCGACTACAGACA-3´ R: 5´-TTAGACCATTCTCCTCAGGAG-3´	645	OR103225
RPL11	D: 5´-ATGGCGCGTGTACCACCG-3´ R: 5´-TTACTTTTTGCTGTTAAGGATGA3-´	549	OR103226
UCP	D: 5´-ATGAAGAAATGTTCGTCAGTG-3´ R: 5´-CTATACTTCTGTATCAACCTCATTAC-3´	819	OR103227

**Figure 5:**
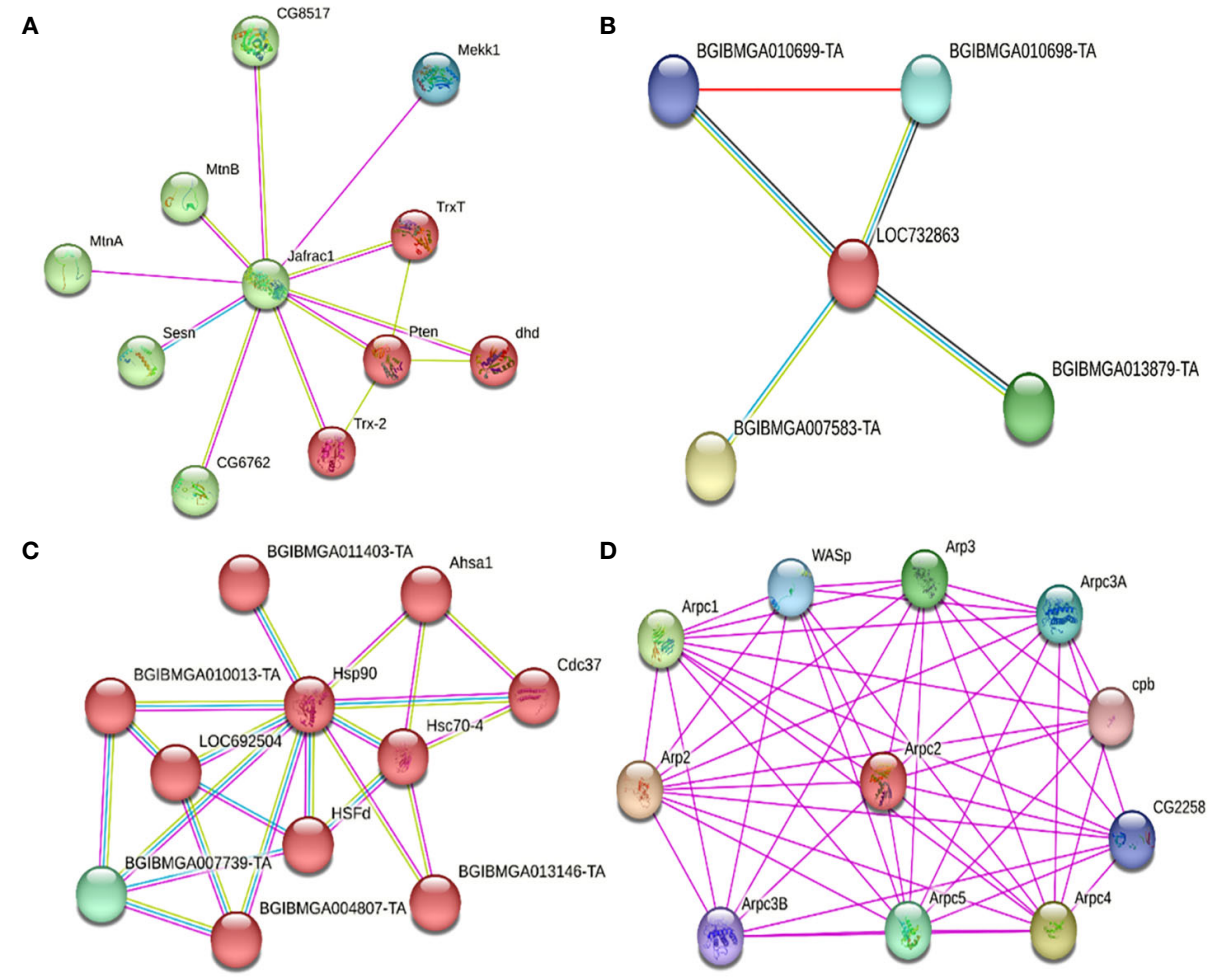
Interactions of different down-regulated and up-regulated proteins due to the primary infection of the baculovirus SfNPV-Ar with its host *S. frugiperda*. **(A)** Interactions of the PRX protein (Jafrac1) with two main clusters. In red, cluster 1, related to cellular redox homeostasis, oxidoreductase, and antioxidant disulfide isomerase activity. In green, cluster 2, related to phosphatidylinositol 3-kinase signaling. **(B)** Interactions of FABP (LOC732863) with proteins related to histone deacetylation, purine base synthesis (BGIBMGA013879-TA) (green), and down-regulation of the JNK pathway (BGIBMGA007583-TA) (yellow). Located in the histone deacetylation complex and the expanded Rpd3L complex. **(C)** Interactions of HSP90 (Hsp90) with two main clusters. In red, cluster 1, related to protein folding and stress response. In green, cluster (2), related to the folding of proteins, acting as chaperones, and having a probable histidine kinase action. **(D)** Interactions of ARP 2/3 (Arpc2) with proteins related to actin polymerization, nucleation, up-regulation of filament reorganization related to the cell cycle (Arp3 and Arpc1), and endocytosis. Located in the ARP 2/3 protein complex, in the cytoskeleton, and actin filaments.

Possible protein-protein interactions were established *in silico*, derived from the differentially expressed transcripts, and using the STRING database system. Once these were identified, their clustering allowed to classify these transcripts according to the following functions: a) apoptosis regulators, b) cytoskeleton regulators, c) protein folding, d) metabolism regulators, e) cellular transport, and f) transcription and translation regulators (**Table 6**). Within the apoptosis regulating proteins, PRX, FABP, and HSP90 were identified. For PRX (Jafrac1, **Figure 5A**), it was estimated to interact with two protein clusters. Cluster 1 (red) related to reactive oxygen species (ROS) in homeostasis, with disulfide isomerase activity, oxidoreductases, and antioxidants. Cluster 2 (green) was associated with phosphatidylinositol 3-kinase signaling. For FABP (LOC732863, **Figure 5B**), it was found related to proteins associated with histone deacetylation, purine base synthesis (BGIBMGA013879-TA) (green), and down- regulation of the JNK pathway (BGIBMGA007583-TA) (yellow). For HSP90 (Hsp90, **Figure 5C**), it interacts with two clusters. Cluster 1 (red) interacts with proteins related to protein folding, stress response, and infection proliferation, while its interaction with Cluster 2 (green) is related to protein folding, acting as chaperones, and histidine kinase action (interaction of Hsp90 with BGIBMGA007739-TA). Within the cytoskeleton regulators, ARP 2/3 (Arpc2, **Table 6**, **Figure 5D**) interacted with a protein cluster related to actin polymerization, nucleation, endocytosis, and cell cycle regulation (interaction between Arp3 and Arpc1).

**Table 6:** Protein-protein interaction regulated by the infection of SfNPV baculovirus on *S. frugiperda* larvae.

Hours post-infection	Affected system	Transcript (Uniprot code)	Regulation	Protein-protein interaction	Function of proteins from clusters with identified interaction
6 hpi	Apoptosis	Peroxiredoxin (Jafrac1)	D-R	Cluster 1: BGIBMGA007947-TA, BGIBMGA008120-TA, BGIBMGA006941-TA, LOC692981, Nucleoredoxin-like, BGIBMGA004183-TA, BGIBMGA006070-TA, BGIBMGA006071-TA. Cluster 2: BGIBMGA009071-TA, BmDJ-1beta.	Cluster 1: Related with homeostasis redox and antioxidant activity, oxidorreductases and disulfide oxidorreductasa. Cluster 2: PI3K signaling
		Fatty acid binding protein (LOC732863)	U-R	Cluser 1: BGIBMGA007583-TA, BGIBMGA013879-TA, BGIBMGA010698-TA, BGIBMGA010699-TA.	JNK pathway down-regulation proteins, activate apoptosis, histone deacetylation, regulate gene expression
	Cytoskeleton	Actin related protein 2/3 complex subunit 2 (Arpc2)	U-R	Cluster 1: Arp2, Arpc4, Arpc1, Arp3, Arpc5, Arpc3A, WASp, CG2258, Arpc3B, Cpb.	Actin polymerization proteins, nucleation, and cell cycle regulation.
	Protein folding	Protein disulfide isomerase (A0A2A4JF99)	D-R	Cluster 1: A0A2A4IV01, A0A2A4J7I0, A0A2A4K368, A0A2A4K4Y6, A0A2A4JMM6. Cluster 2: A0A2A4IUV4, A0A2A4J7L8, A0A2A4IVA2, A0A2A4IYI5. Cluster 3: A0A2A4JVI3.	Cluster 1: Proteins involved in other protein folding (chaperons). Cluster 2: Protein involved in the formation of disulfide bonds. Cluster 3: Eukaryotic elongation factor.
	Lipid metabolism and transport	AcylCoA binding protein (ACBP-like)	U-R	Cluster 1: Scpx, BGIBMGA011144-TA, LOC692961, BGIBMGA006608-TA, BGIBMGA004502-TA, LOC732974, BGIBMGA003725-TA.	Labeling, transport, and import of proteins into the peroxisome.
	Transcription and translation	60S ribosomal protein L19 (A0A2A4IZU1)	D-R	Cluster 1: RpS20, A0A2A4ISJ8, A0A2A4IUE9, A0A2A4IUS3, A0A2A4IY20, A0A2A4IY56, A0A2A4IZB4, A0A2A4J0J0, A0A2A4J2L9, A0A2A4J3P1.	Activation of translation and ribosome assembling
		Ribosomal protein L11 (RpL11)	U-R	Cluster 1: RpL37, RpS2e, RpL31, RpL27A, RpL21, RpS21, RpS15, RpS17, Ubi3.	Ribosomal components and translation system
12 hpi	Apoptosis	Fatty acid binding protein (LOC732863)	D-R	Cluster 1: BGIBMGA007583-TA, BGIBMGA013879-TA, BGIBMGA010698-TA, BGIBMGA010699-TA.	JNK pathway down-regulation able to activate apoptosis, histone deacetylation, gene expression regulation
	Cytoskeleton	Actin related protein 2/3 complex subunit 2 (Arpc2)	U-R	Cluster 1: Arp2, Arpc4, Arpc1, Arp3, Arpc5, Arpc3A, WASp, CG2258, Arpc3B, Cpb.	Actin polymerization, nucleation, and cell cycle regulation
	Protein folding	Protein disulfide isomerase (A0A2A4JF99)	D-R	Cluster 1: A0A2A4IV01, A0A2A4J7I0, A0A2A4K368, A0A2A4K4Y6, A0A2A4JMM6. Cluster 2: A0A2A4IUV4, A0A2A4J7L8, A0A2A4IVA2, A0A2A4IYI5. Cluster 3: A0A2A4JVI3.	Cluster 1: Protein folding acting as chaperon. Cluster 2: disulfide bond formation. Cluster 3: Eukaryotic elongation factor.
	Lipid metabolism and transport	ATP synthase subunit Omitochondrial (A0A2A4J874)	U-R	Cluster 1: A0A2A4IV62, A0A2A4JHT3, A0A2A4JL11, A0A2A4K6C5, A0A2A4J1Q8. Cluster 2: A0A2A4JHC9, A0A2A4JAN6, A0A2A4JHJ2, A0A2A4JM46, A0A2A4JR94.	Cluster 1: ATP synthesis and proton transport. Cluster 2: ATP synthesis and oxidative phosphorylation.
	Transcription and translation	Ribosomal protein L7 (RpL7)	D-R	Cluster 1: RpL32, RpL37, RpS2e, RpL31, RpL27A, RpL21, RpS17, RpL4, RpL36, Ubi3.	Cluster 1: mRNA translation.
24 hpi	Apoptosis	Fatty acid binding protein (LOC732863)	D-R	Cluster 1: BGIBMGA007583-TA, BGIBMGA013879-TA, BGIBMGA010698-TA, BGIBMGA010699-TA.	JNK pathway down-regulation able to activate apoptosis, histone deacetylation, gene expression regulation
	Cytoskeleton	Actin related protein 2/3 complex subunit 2 (Arpc2)	D-R	Cluster 1: Arp2, Arpc4, Arpc1, Arp3, Arpc5, Arpc3A, WASp, CG2258, Arpc3B, Cpb.	Actin polymerization, nucleation, and cell cycle regulation.
		Acidic leucine rich nuclear phosphoprotein 32 (Anp32a)	U-R	Cluster 1: EVAR_85023_1, Xpo1, ELAVL4, PCNA, DMAP1, XPO5, XPO5-2, EVAR_74068_1, xpo6-b, XPO6.	Export, transport, and relocation of proteins, and regulation of stress response.
	Protein folding	HSP90 (Hsp90)	U-R	Cluster 1: Cdc37, Ahsa1, BGIBMGA011403-TA, BGIBMGA010013-TA, HSFd, BGIBMGA013146-TA, BGIBMGA004807-TA, Hsc70-4, LOC692504. Cluster 2: BGIBMGA007739-TA.	Cluster 1: Folding, stress response, and viral infection proliferation. Cluster 2: Chaperones and histidine kinases.
	Transcription and translation	Ribosomal protein L7 (RpL7)	D-R	Cluster 1: RpL32, RpL37, RpS2e, RpL31, RpL27A, RpL21, RpS17, RpL4, RpL36, Ubi3.	Cluster 1: mRNA translation.
		60S ribosomal protein L19 (A0A2A4IZU1)	U-R	Cluster 1: RpS20, A0A2A4ISJ8, A0A2A4IUE9, A0A2A4IUS3, A0A2A4IY20, A0A2A4IY56, A0A2A4IZB4, A0A2A4J0J0, A0A2A4J2L9, A0A2A4J3P1.	Translation activation and ribosome assembling.

U-R, Up-regulated; D-R, Down-regulated.

**Figure 6:**
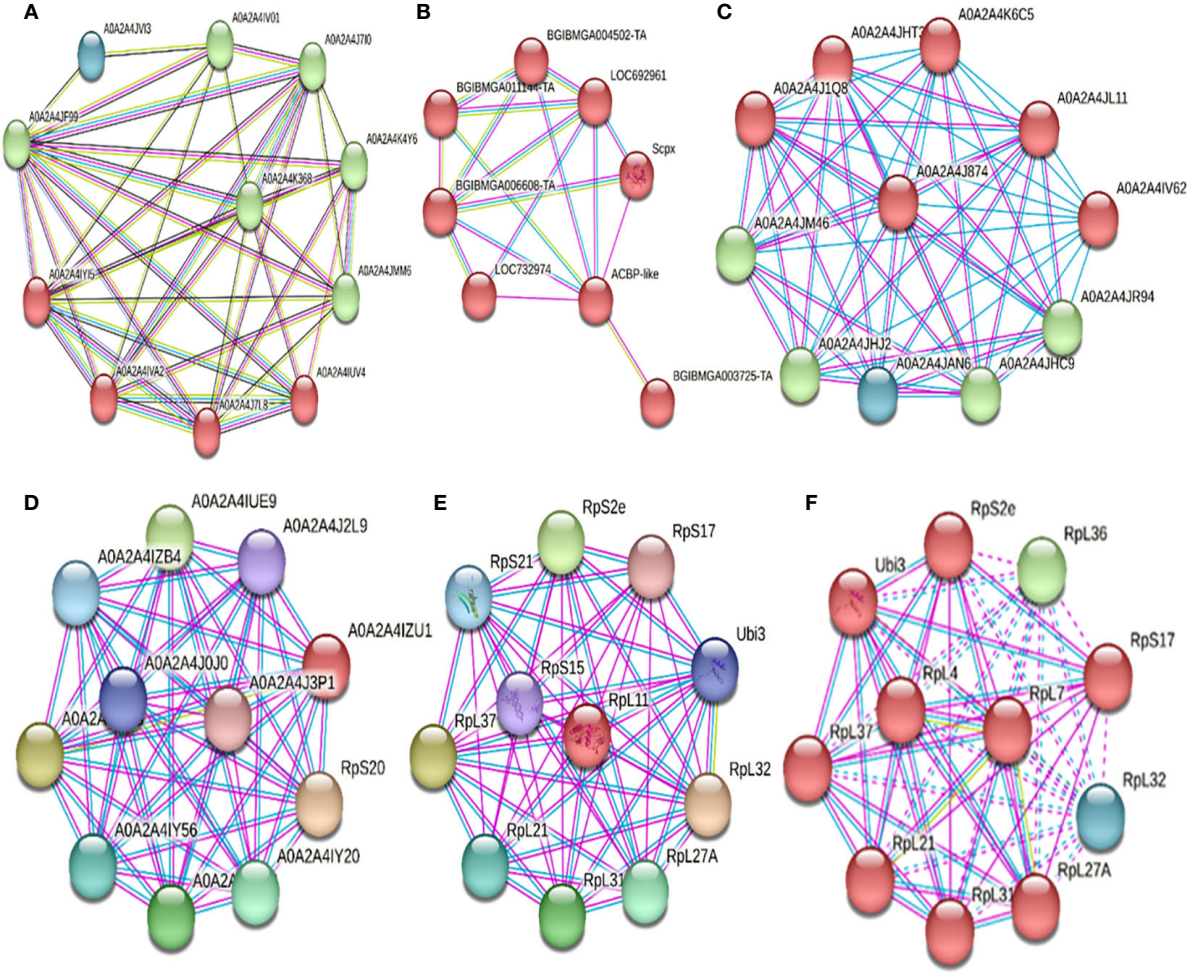
Interactions of various proteins either down-regulated or up-regulated due to the infection of the baculovirus SfNPV-Ar with its host *S. frugiperda*. **(A)** Interactions of PDI (A0A2A4JF99) with three main clusters. In green, cluster 1, interaction with proteins involved in protein folding and chaperones. In red, interaction with proteins related to protein folding and the formation of disulfide bonds. In blue, interaction with a eukaryotic elongation factor. All interactions were physically located in the endoplasmic reticulum. **(B)** Interactions of AcilBP (ACBP-like) with proteins related to tagging, transport, and import of proteins to the peroxisome, with this protein (AcilBP) being an acyltransferase, located on the peroxisome membrane. **(C)** Interactions of the ATPS0 protein (A0A2A4J874) with two main protein clusters. In red, cluster 1, with proteins related to ATP synthesis in the mitochondrial proton transport complex in the ATP synthase complex. In green and blue, cluster 2, related to ATP synthesis and oxidative phosphorylation in the ATP synthase complex. Both interactions are in the mitochondria. **(D)** Interactions of RPL19 (A0A2A4IZU1) with proteins related to mRNA translation and the assembly of the large ribosomal subunit. Located in the ribosome in the cytoplasm. **(E)** Interactions of RPL11 (RpL11) with proteins that are part of the ribosomes, and these also form part of the translation system. They are physically located in the cytosol in the ribosomes. **(F)** Interactions of RPL7 (RpL7) with proteins that are part of the ribosomes, and these also form part of the translation system. They are physically located in the large subunit of the ribosomes.

For protein folding regulators, PDI and HSP90 were interfering (**Table 6**). For PDI (A0A2A4JF99, **Table 6**, **Figure 6A**), it interacted with three clusters: the first (Cluster 1, green) and second (Cluster 2, red) related to protein folding, chaperones, and disulfide bond formation, respectively. Cluster 3 (blue) is related to a eukaryotic elongation factor. All interactions were located physically in the endoplasmic reticulum. For HSP90 (Hsp90, **Table 6**, **Figure 6A**), upon interaction with Cluster 1 proteins, it was determined to participate in protein folding and stress response. For metabolism and cellular transport regulators, AcilBP and ATPS0 were interfering (**Table 6**). For AcilBP (ACBP-like, **Table 6**, **Figure 6B**), it interacted with proteins marking and transporting to the peroxisome, while ATPS0 (A0A2A4J874, **Table 6**, **Figure 6C**) interacted with two main protein clusters. Cluster 1 (red) includes proteins related to ATP synthesis in the mitochondrial proton transport complex of ATP synthase. Cluster 2 (green and blue) is related to ATP synthesis and oxidative phosphorylation in the ATP synthase complex. Both interactions are in the mitochondria. For transcription and translation regulators, RPL19, RPL11, RPL7, and FPN32 were interfering (**Table 6**). For RPL19 (A0A2A4IZU1, **Table 4**, **Figure 6D**) and RPL11 (RpL11, **Table 6**, **Figure 6E**), they interact with ribosome proteins promoting assembly and participate in mRNA translation. For RPL7 (RpL7, **Table 6**, **Figure 6F**), it interacts with a protein cluster responsible for mRNA translation. In the case sequences of MEP20, MG17, and UCP, did not yield significant identities in the STRING system, so their interactions were not evaluated in this study.

## 4 Discussion

Gene regulation during the first stages of infection of a baculovirus to the midgut epithelium of the fall armyworm, *S. frugiperda*, was studied by transcriptomics and interactomics analyses of their protein products. We detected changes in gene expression during the primary infection of the SfNPV-Ar baculovirus at 6, 12, and 24 hpi.

### 4.1 The apoptotic system implications

Interference on apoptosis, cytoskeleton, protein folding, metabolism, and ribosome structure was detected since 6 hpi. Regarding apoptosis, a down-regulation of the PRX gene was detected, which is believed to participate with proteins related to redox homeostasis, which may have an antioxidant effect and oxidoreductase activity (Vessaro-Silva et al., 2019). It is also believed to participate in phosphatidylinositol 3 kinase (PI3K) signaling. This has been previously described by Wu et al. (2009); Yu et al. (2016), and Huang et al. (2018), who in the early post-infection hours detected an increase in ROS. As for PI3K, Pinzón et al. (2009) mention that this protein is related to the shutting down of pro-apoptotic systems, so the down- regulation of PRX might be implicated in a cellular strategy of the FAW to turn off survival system regulators and induce an increase in ROS. In relation to this, the up-regulation of FABP at 6 hpi, and its subsequent down-regulation at 12 and 24 hpi, may influence the JNK system involved in regulating apoptotic signals (Lin, 2003). This can indicate a cellular defense in insects against viral infection in the early stages, demonstrating the action of an apoptosis regulation system in the early post-infection stages. Also, Xue et al. (2012) detected the expression of p35 at 6 hpi, and since it is considered an anti-apoptotic protein (Sahdev et al., 2010), it would prevent the possible effect of the down-regulation of PRX and up-regulation of FABP.

The down-regulation of FABP was detected at this post-infection time. Given that this protein is related to the JNK pathway and apoptotic signaling at 6 hpi, its inhibition at 12 hpi could be correlated with the expression of viral anti-apoptotic genes *iap1* and *iap2* (Xue et al., 2012). This deactivation might prevent apoptosis in the insect. On the other hand, at 24 hpi, alterations also detected the regulation of apoptosis, the cytoskeleton, protein folding, as well as transcription and translation. The down-regulation of FABP was detected, as it was observed at 12 hpi. FABP is involved in the regulation of the JNK pathway, as previously described, suggesting that this transcript might be suppressing the apoptotic signal in insects during intermediate and late stages of viral infection. Our results in the interactomic analysis indicated that FABP is related with the JNK pathway, through the interaction with BGIBMGA007583-TA and BGIBMGA000461-TA (data not shown), which down-regulate this pathway. Lin (2003) mentions that the JNK pathway both up- and down-regulates the apoptosis, as this pathway is dependent of a complex series of stimuli. According to Xue et al. (2012) and Nguyen et al. (2013), they also detected the shutdown of genes related to apoptosis and stress at 24 hpi. Furthermore, Shrestha et al. (2019) reported a shutdown in the expression of genes related to cellular defense between 12 and 24 hpi.

### 4.2 Effects on the cytoskeleton

Regarding cytoskeleton regulation, the up-regulation of ARP 2/3 was detected, which could be related to up-regulation of the cell cycle and cytoskeleton reorganization. This possible effect is supported by Swali et al. (2011) who mention that ARP 2/3 is essential for cell cycle development. However, other authors (Goley et al., 2006; Xue et al., 2012; Nguyen et al., 2013; Mu et al., 2016) mention that the accumulation of ARP 2/3 in the nucleus is vital for the correct replication of baculoviruses and nucleocapsid assembly; and Huang et al. (2018) indicate that the rearrangement of the cytoskeleton begins in the early stages of baculovirus infection. A gradual increase in ARP2/3 transcripts from 6 to 12 hpi was observed, but later at 24 hpi the gene transcripts seems to be down-regulated (**Figure 4**). This shutdown in the later stages of infection might indicate the damage onset to the insect cell structure. A similar phenomenon was reported by Bao et al. (2009) and Mu et al. (2016), who mentioned that between 6-12 hpi, cytoskeletal rearrangement and cell cycle arrest in the insect are essential for the formation of nucleocapsids and viral DNA replication. This is consistent with the up-regulation of ARP 2/3 observed at 6 and 12 hpi in this study, but this will have to be experimentally verified. By 24 hpi, maintaining cellular integrity is no longer necessary, so the virus could repress its expression during these post-infection times.

### 4.3 Protein folding regulation

PDI, a protein involved in protein folding and the transcripts was found to be down regulated during 6 and 12 hpi; the down regulation of transcripts was higher in 6 hpi in comparison to 12 hpi (**Figure 4**). This protein interferes in the regulation of the NADPH oxidase system and its potential as a source of ROS. Hence, it is inferred that the virus could suppresses its expression to prevent ROS production and cellular stress. However, this down-regulation of the PDI gene, could be involved in the formation of disulfide bonds, protein folding, chaperones, and possibly as an elongation factor. When PDI is normally expressed in the insect, it can activate the NADPH oxidase system (Fernandes et al., 2009), changing ROS concentrations, so its shutdown could affect protein folding. Li et al. (2022) detected a shutdown of immunity genes at 6 hpi, so it can be inferred that if PDI is down- regulated, the activation of NADP oxidase could be prevented, lowering ROS concentrations in infected cells. As for the protein folding at 24 hpi, the up-regulation of the HSP90 transcript was detected, a protein known to be involved in this process and as a chaperone protein. However, it has also been linked to the UPR system (Koczka et al., 2018) and in promoting viral infection proliferation (Mao et al., 2021). These two phenomena are correlated. Koczka et al. (2018) stated that in the late stages of viral infection, there is an up-regulation of genes related to chaperones and heat shock proteins, aiming to counteract the UPR system. On the other hand, Mao et al. (2021) indicated that the up-regulation of HSP70, which interacts with HSP90, promotes protein folding and the proliferation of the viral infection. This phenomenon also assists in apoptosis regulation in advanced infection stages and could relate to the virus attempt to keep the cell viable in advanced infection stages by regulating the UPR system (Fortugno et al., 2003).

### 4.4 Metabolism, ribosome structure and cellular transport

In terms of metabolism and cellular transport, this study identified the up-regulation of the AcilBP transcript, associated with the tagging and transport of proteins to the peroxisome. This could potentially disrupt the peroxisome balance, hampering communication with the mitochondria, destabilizing the redox equilibrium, and causing cellular damage (Wang et al., 2013). Regarding the ribosome structure, this study noted the down-regulation of RPL19 at 6 hpi, which could impact its assembly and hinder protein translation in insects (Huang et al., 2018; Li et al., 2022). Also, related to ribosome functionality, up-regulation of RPL11 was detected at 6 hpi. This protein is known to interact with other proteins involved in translation. A similar phenomenon was observed by Xue et al. (2012), who at 6 hpi, noted an increase in genes related to ribosomes and, consequently, to translation, but in the silkworm *Bombyx mori*. This suggests a viral strategy to promote gene expression keeping insect cells functionality (Xue et al., 2012). This supports the subsequent stages of viral replication and the expression of its molecular machinery. However, the down- regulation of RPL19 aligns with what was described by Hong et al. (2014), who mentioned that the up-regulation of RPL19 causes the pre-activation of the UPR system linked to the activation of apoptotic signals. Therefore, the observed down- regulation in this study may indicate that the activation of this pathway is inhibited in the early stages of infection. Concerning the insect’s basal metabolism, this study identified the up-regulation of the ATPS0 transcript, known to play a role in oxidative phosphorylation processes, which suggests a potential increase in cellular energy requirements at 12 hpi. Nayyar et al. (2017) also observed an uptick in proteins related to metabolism during mid-stages of viral infection, suggesting that this occurred so the insect could meet the energy demands triggered by the viral infection.

### 4.5 Transcription and translations effects

Concerning transcription and translation regulators, this study observed the up-regulation of the FPN32 and RPL19 protein transcripts and the down-regulation of RPL7 at 24 hpi. It is known that FPN32 participates in protein and RNA export from the nucleus, so this protein might assist newly formed viral nucleocapsids in the nucleus to transport to the cell membrane, budding from the columnar cells to the insect’s hemolymph. Nguyen et al. (2013) also reported up-regulation at 24 hpi of genes related to cellular transport. Within the same topic, a down- regulation of the RPL7 transcript was identified at 12 hpi, which is associated with ribosome formation. Xue et al. (2012) and Nguyen et al. (2013) reported similar effects on the regulation of genes that might impact the initiation of transcription, translation, and viral replication at this same post-infection time. This observation could suggest that the repression in RPL7 expression is a cellular strategy by the insect to avoid translating viral mRNA. Similarly, Bao et al. (2009) determined that the onset of the production of budding virions occurs between 12 and18 hpi, and their discharge from the cell at 24 hpi, concurrent with the up-regulation of FPN32 reported in this study. In relation with the up-regulation of the RPL19 transcript, which is known to participate in ribosome assembly and mRNA translation, the virus could use this strategy to help the expression of its late genes. According to Xue et al. (2012) and Nguyen et al. (2013), they reported up-regulation of translation-related genes at 24 hpi. However, Hong et al. (2014) showed that the up-regulation of RPL19 is linked to the pre-activation of the UPR system. As mentioned before, RPL19 might play another role related to apoptosis in the late stages of viral infection. Finally, also at 24 hpi, down-regulation of RPL7 was observed, which is involved in ribosome formation. Earlier reports (Xue et al., 2012; Nayyar et al., 2017; Shrestha et al., 2019) stated that in the late stages of the primary infection, there is down-regulation of genes related to cellular stability and metabolism due to the cessation of cellular processes. Thus, RPL7 might influence a shutdown of the mRNA translation system in the advanced stages of cellular infection, leading to the cessation of the viability of the insect’s tissues.

In general, data obtained in this work shows how complicated is the mechanism used by the virus to start the infection of the whole insect body, by redirecting the normal host metabolism of the initial infection. Clearly, this type of studies should be eventually supported by direct evidence of the effects of shutting-down of turning-on specific host genes, which will be focused on future research.

## 5 Conclusions

The results obtained in this study could indicate that the virus causes significant alterations in the gene expression process during the insect’s early infection stages. However, in the initial hours post-infection, the virus apparently protects the integrity of the infected cells and prevents them from undergoing apoptosis, ensuring its replication. The virus also apparently induces a rearrangement of the insect cell cytoskeleton, with the up-regulation of the ARP 2/3 protein transcripts at 6 to 12 hpi and the repression of this protein expression at 24 hpi. Additionally, the virus regulates protein folding, evident by the shutting down of PDI between 6 and 12 hpi and the up-regulation of HSP90 at 24 hpi, elevating cellular metabolism with the up-regulation of AcilBP at 6 hpi and ATPS0 at 12 hpi. Also, the virus can control the insect’s transcription and translation processes, through the down-regulation of RPL19 and up-regulation of RPL11 at 6 hpi, the down-regulation of RPL7 at 12 and 24 hpi, and the up-regulation of RPL19 and FPN32 at 24 hpi. Through these actions, the SfNPV-Ar virus can take over the cellular machinery and deactivate the host defense systems as early as 6 hpi.

This study determined which transcripts and proteins are responsible for the virulence of a highly efficient baculovirus strain, potentially useful for controlling the FAW. This knowledge may have some impact by optimizing and selecting the best baculovirus strains for its use in biological control programs against this pest, thereby reducing the devastation it causes in global maize production.

## Data availability statement

The datasets presented in this study can be found in online repositories. The names of the repository/repositories and accession number(s) can be found below: https://www.ncbi.nlm.nih.gov/genbank/, OR103221 https://www.ncbi.nlm.nih.gov/genbank/, OR103219 https://www.ncbi.nlm.nih.gov/genbank/, OR103216 https://www.ncbi.nlm.nih.gov/genbank/, OR103217 https://www.ncbi.nlm.nih.gov/genbank/, OR103222 https://www.ncbi.nlm.nih.gov/genbank/, OR103218 https://www.ncbi.nlm.nih.gov/genbank/, OR103223 https://www.ncbi.nlm.nih.gov/genbank/, OR103224 https://www.ncbi.nlm.nih.gov/genbank/, OR103220 https://www.ncbi.nlm.nih.gov/genbank/, OR103225 https://www.ncbi.nlm.nih.gov/genbank/, OR103226 https://www.ncbi.nlm.nih.gov/genbank/, OR103227.

## Ethics statement

The manuscript presents research on animals that do not require ethical approval for their study.

## Author contributions

JR-N: Conceptualization, Data curation, Formal Analysis, Investigation, Methodology, Software, Validation, Visualization, Writing – original draft, Writing – review and editing. JI: Conceptualization, Data curation, Formal Analysis, Writing – original draft, Writing – review and editing. MR-C: Conceptualization, Formal Analysis, Funding acquisition, Investigation, Methodology, Project administration, Resources, Supervision, Validation, Writing – original draft, Writing – review and editing.

## Glossary

**Table:**

AcilBP	Acyl-CoA binding protein
ARP 2/3	Actin-related protein 2/3 complex subunit 2
ATPSO0	ATP synthase subunit O mitochondrial
D-R	Down-regulated
ds cDNA	Double stranded complementary DNA
FABP	Fatty acid binding protein
FAW	Fall armyworm
FPN32	Acidic leucine rich nuclear phosphoprotein
hpi	Hours post infection
HB	Hybridization buffer
HSP90	Heat shock protein 90
LC_50_	Mean lethal concentration
MEP20	Muscular protein 20
MG17	MG17 protein
OBs	Occlusion bodies
ORF	Open reading frame
PDI	Disulfide isomerase
PRX	Peroxiredoxin
ROS	Reactive oxygen species
RPL11	Ribosomal protein L11
RPL19	60S ribosomal protein L19
RPL7	Ribosomal protein L7
rpm	Revolutions per minute
SDW	Sterile distilled water
SfNPV-Ar	*Spodoptera frugiperda* nuclear polyhedron virus strain Ar
ss cDNA	Single stranded complementary DNA
UCP	Uncharacterized protein
UPR	Unfolded protein response
U-R	Up-regulated
